# Baseline functional connectivity may predict placebo responses to accelerated rTMS treatment in major depression

**DOI:** 10.1002/hbm.24828

**Published:** 2019-10-21

**Authors:** Guo‐Rong Wu, Xiaowan Wang, Chris Baeken

**Affiliations:** ^1^ Key Laboratory of Cognition and Personality, Faculty of Psychology Southwest University Chongqing China; ^2^ Department of Psychiatry and Medical Psychology Ghent University Ghent Belgium; ^3^ Department of Psychiatry, Vrije Universiteit Brussel (VUB) Universitair Ziekenhuis Brussel (UZBrussel) Brussels Belgium; ^4^ Ghent Experimental Psychiatry (GHEP) Lab Ghent University Ghent Belgium; ^5^ Department of Electrical Engineering Eindhoven University of Technology Eindhoven The Netherlands

**Keywords:** depression, functional connectivity, placebo responses, rostral ACC, sham iTBS, sham rTMS

## Abstract

Although in theory sham repetitive transcranial magnetic stimulation (rTMS) has no inherent therapeutic value, nonetheless, such placebo stimulations may have relevant therapeutic effects in clinically depressed patients. On the other hand, antidepressant responses to sham rTMS are quite heterogeneous across individuals and its neural underpinnings have not been explored yet. The current brain imaging study aims to detect baseline neural fingerprints resulting in clinically beneficial placebo rTMS treatment responses. We collected resting‐state functional magnetic resonance imaging data prior to a registered randomized clinical trial of accelerated placebo stimulation protocol in patients documented with treatment‐resistant depression (http://clinicaltrials.gov/show/NCT01832805). In addition to global brain connectivity and rostral anterior cingulate cortex (rACC) seed‐based functional connectivity (FC), elastic‐net regression and cross‐validation procedures were used to identify baseline intrinsic brain connectivity biomarkers for sham‐rTMS responses. Placebo responses to accelerated sham rTMS were correlated with baseline global brain connectivity in the rACC/ventral medial prefrontal cortex (vmPFC). Concerning the rACC seed‐based FC analysis, the placebo response was associated positively with the precuneus/posterior cingulate (PCun/PCC) cortex and negatively with the middle frontal gyrus. Our findings provide first brain imaging evidence for placebo responses to sham stimulation being predictable from rACC rsFC profiles, especially in brain areas implicated in (re)appraisal and self‐focus processes.

## 1 INTRODUCTION

Placebos are sham medical or surgical treatments resulting in substantial beneficial effects on clinical outcome (Ashar, Chang, & Wager, 2017). However, by definition, it should be ineffective or not specifically effective for the condition being treated (Beauregard, 2007). Indeed, the psychophysiological responses elicited by placebos can be very specific depending on the provided information, guided by subjective factors such as clinical expectations, beliefs, and hope for improvement (Beauregard, 2009). Clinically effective placebo responses have been observed in the antidepressant therapy with pharmacological and nonpharmacological interventions (Baeken, Wu, & van Heeringen, 2019; Brunoni, Lopes, Kaptchuk, & Fregni, 2009; Razza et al., 2018; Sikora et al., 2016; Weimer, Colloca, & Enck, 2015).

Repetitive transcranial magnetic stimulation (rTMS) is a noninvasive neuromodulation therapy approved by FDA for treatment of depressed patients who do not benefit fully from regular antidepressant pharmacotherapy. Although the placebo response to rTMS treatment seems to be of a similar magnitude as those of psychopharmacotherapy (Brunoni et al., 2009), recent work suggests that such placebo effects may be part of the clinical efficacy of rTMS in depressed patients (Baeken, Wu, & van Heeringen, 2019; Razza et al., 2018). Notwithstanding, the neurobiological underpinnings of sham rTMS explaining treatment response—even in treatment‐resistant depressed patients—are still not fully characterized. Having better insights into the beneficial mechanisms of the sham neurostimulation could help to guide the therapeutic outcome.

Neuroimaging methods may have the potential for unraveling the phenomenon behind the placebo response of neurostimulation treatment protocols. Indeed, it has been suggested that placebo responses correlate with changes in a core network of brain regions associated with self‐evaluation, social cognition, future thinking, and the evaluation of rewards and punishment (Ashar et al., 2017). Furthermore, positron emission tomography (PET) and electroencephalography (EEG) assessment provide a strong link between the improvement in depressive symptoms and the neural activity in the anterior and posterior cingulate cortices (Leuchter, Cook, Witte, Morgan, & Abrams, 2002; Mayberg et al., 2002; Pecina & Zubieta, 2015). A recent meta‐analysis also highlights the default mode network (DMN) playing a critical role in placebo treatment (Ashar et al., 2017). Furthermore, baseline rostral anterior cingulate cortex (rACC) activity/connectivity may be a predictor of depression alleviation (Pizzagalli, 2011; Pizzagalli et al., 2018; Whitton et al., 2019). Given the heterogeneity of depressive symptoms, the identification of a pretreatment neural biomarker could contribute a more optimally personalized treatment choice, even for clinical meaningful placebo responses to neurostimulation.

In order to increase our insight into the placebo responses of sham rTMS treatment in depressed patients, for the current project resting‐state fMRI (rs‐fMRI) scans were collected prior to neurostimulation. In this research set‐up, we applied a specific form of rTMS parameters, called accelerated intermittent theta‐burst stimulation (iTBS), where instead of the daily single stimulations, to reduce the total stimulation period multiple sessions per day are applied in a couple of days instead of weeks (Chung, Hoy, & Fitzgerald, 2015). It has been stated that such accelerated protocols are possibly more sensitive to placebo responses (Baeken, 2018). To examine these placebo responses on the brain level, we performed a global brain connectivity (GBC) analysis on the baseline rs‐fMRI data. The GBC is a data‐driven approach and particularly well‐suited to identify connectivity alterations that might be missed by seed‐driven approaches (Martuzzi et al., 2011). Based on the established hypotheses, the rACC seed‐based FC was also computed to examine possible predictors of the sham iTBS treatment response. In line with prior pharmacotherapy placebo trials, we hypothesized that placebo responses to sham accelerated iTBS (aiTBS) would be predicted by rACC connectivity, including both the global brain connectivity and seed‐based FC.

## 2 MATERIALS AND METHODS

### 2.1 Participants

The detailed demographic data and exclusion criteria of this registered trial (Theta Burst Study Ghent [TBS Ghent] http://clinicaltrials.gov/show/NCT01832805) are available in Duprat et al. (2016). In short, the Mini‐International Neuropsychiatric Interview was used to establish the diagnoses of major depression (Sheehan et al., 1998), and patients were at least Stage I treatment‐resistant (Rush, Thase, & Dubé, 2003). After a psychotropic washout period, all patients were at least 2‐week drug‐free before the start of the stimulation protocol. In this study, we only focused on the 22 (16 females; mean age = 43, standard deviation [SD] = 12.24, 18–61 years) right‐handed treatment‐resistant depressed (TRD) patients who were randomized to receive first the sham aiTBS. All participants gave written informed consent and the study was approved by the local ethics committee of the Ghent University. All patients were naïve to rTMS treatment. This study was part of a larger research on the neurobiological effects of aiTBS in depressed patients (Baeken, Duprat, Wu, De Raedt, & van Heeringen, 2017).

### 2.2 Stimulation protocol

A Magstim Rapid^2^ Plus^1^ magnetic stimulator (Magstim Company Limited, Wales, UK) connected to a sham figure of eight‐shaped cooled coil was used to perform sham aiTBS. This specifically designed sham coil is identical to an active coil, provides the same auditory stimuli, but has no active stimulation. The localization of the stimulation site (left DLPFC, that is, the center part of the midprefontal gyrus [Brodmann 9/46]), the BrainSight neuronavigation system (Brainsight™, Rogue Resolutions, Inc, New York, NY) was guided by structural magnetic resonance imaging (MRI). The protocol comprising in total 20 sham iTBS sessions was spread across the four succeeding days at five daily sessions, within a 1‐week period. In each session, patients received 1,620 pulses (110% of the individual resting motor threshold) in 54 triplet bursts with train duration of 2 s and an 8‐s cycling period. There was a time gap (approximately 15 min) between two sequential sessions. During the entire stimulation protocol, patients wore earplugs and were blindfolded.

### 2.3 Clinical assessment

The 17‐item Hamilton Depression Rating Scale (HDRS; Hamilton, 1967) was used to assess depression severity at prestimulation (HDRS_baseline_) and after a week of sham aiTBS treatment (HDRS_post_). These raters were unaware of the actual treatment that was provided. To have an idea of the magnitude of the placebo response, the percent reduction in HDRS scores from baseline to the end of the sham aiTBS treatment period (HDRS_%change_ = [HDRS_baseline_ – HDRS_post_]/HDRS_baseline_) was calculated.

### 2.4 Neuroimaging data acquisition

Scanning was performed on a 3T Siemens Tim Trio MRI scanner (Siemens Medical Solutions, Erlangen, Germany) with a 32‐channel head coil before the aiTBS therapy trial. High‐resolution anatomical images were collected using a MPRAGE sequence (176 sagittal slices; repetition time (TR) = 2,530 ms; echo time (TE) = 2.58 ms; flip angle = 7°; field of view = 220 × 220 mm^2^; voxel size = 0.9 × 0.9 × 0.9 mm^3^). A total of 300 volumes of resting‐state echo planar imaging BOLD data were acquired with the eyes closed condition (TR =2,000 ms; TE = 29 ms; flip angle = 90°; FOV = 192 × 192 mm^2^; voxel size = 3 × 3 × 3 mm^3^; slice thickness = 3.0 mm; slice gap = 1.0 mm).

### 2.5 Data preprocessing

Data processing was performed with the SPM12 (http://www.fil.ion.ucl.ac.uk/spm/) and CONN toolbox (version 18.b; Whitfield‐Gabrieli & Nieto‐Castanon, 2012). Anatomical images were segmented into gray matter, white matter (WM) and cerebrospinal fluid (CSF). The initial five volumes of the functional images were discarded to achieve steady‐state magnetization. The remaining volumes were slice‐timing corrected, spatially realigned, unwrapped, co‐registered with anatomical image, then normalized to MNI space and smoothed with 6‐mm FWHM Gaussian kernel.

A linear regression was applied to remove possible spurious variances from the data (Behzadi, Restom, Liau, & Liu, 2007; Power, Barnes, Snyder, Schlaggar, & Petersen, 2012; Saad et al., 2012), including (i) six motion artifact parameters and their temporal derivatives, (ii) the scrubbing series generated by the Artifact Detection Tool with 97th‐percentile threshold, (iii) nonneuronal sources of noise estimated from unsmoothed data using two separate approaches: (a) the anatomical component correction method (aCompCor, the representative signals of no interest from subject‐specific WM and CSF included the top five principal components from WM and the top five from CSF mask), (b) whole‐brain signal regression (global mean signal, and the averaged signals from subject‐specific WM and CSF mask). Then the residual time series were linearly detrended, temporally despiked with a hyperbolic tangent squashing function, and temporally band‐pass filtered (0.01–0.1 Hz).

### 2.6 Brain connectivity analysis

The functional connectivity analysis were carried out in the CONN toolbox. The preprocessed BOLD signals were submitted to evaluate global brain connectivity (GBC, $\mathrm{GBC}\left(i\right)=\sqrt{\frac{1}{n}\sum_{j}r_{\mathrm{ij}}^{2}}$, where *r*_ij_ is the correlation coefficient between voxels *i* and *j*, *n* = #voxels inside the brain), which is a graph theory‐based connectivity metric assessing network centrality at each voxel (Martuzzi et al., 2011). The GBC is a whole‐brain voxel‐based measure of connectivity, addresses qualitatively different question about brain connectivity than seed‐based analysis. Aberrant GBC might suggest disturbed information processing from a brain region to other brain areas.

**Figure 1:**
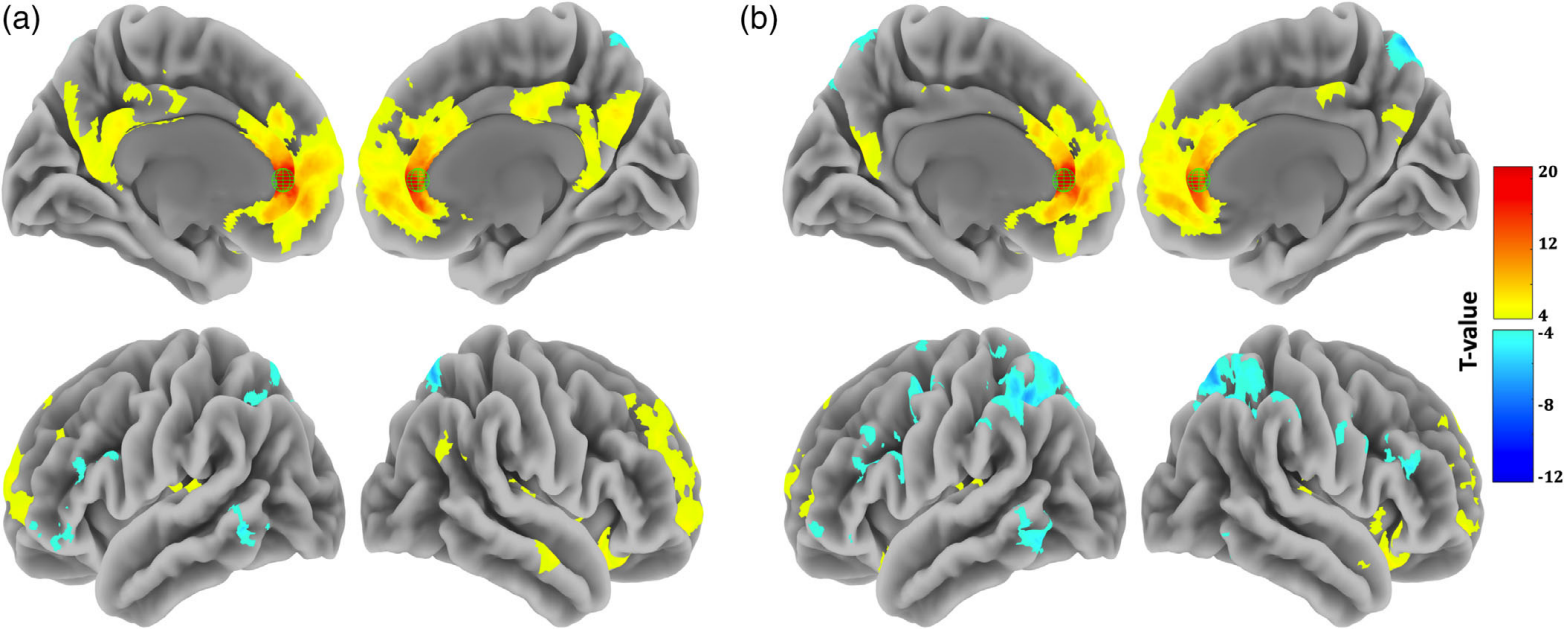
One‐sample *t*‐tests results of the baseline rACC seed‐based FC (*p* < .05, FWE correction at the cluster level with a voxel‐level threshold of *p* < .001 uncorrected). Two confound regression strategies were used: (a) aCompcor and (b) global signal regression

The seed‐based FC maps were obtained by computing the Fisher‐transformed correlation coefficients between the average BOLD signals in rACC and the signals in all other brain voxels. The seed rACC was defined as a 6‐mm‐diameter sphere centered on a point with MNI coordinates (*x* = 0, *y* = 38, *z* = 4), based on the coordinate from a previous placebo study of open‐label antidepressant medication treatment (Sikora et al., 2016). The group rACC seed‐based FC map was created by performing a random effects one‐sample *t*‐test across all participants (Figure 1), with age, gender and mean framewise displacement (FD) as nuisance covariates.

### 2.7 Predicting placebo effects

We conducted elastic‐net regression to select the minimal set of voxels that best predicted depression symptom improvement, implementing in the machine learning toolbox scikit‐learn (version 0.19.0; Pedregosa et al., 2011). To overcome over‐fitting, the leave‐one‐out cross‐validation was used. The GBC maps were entered as independent variable for the prediction of HDRS_%change_ scores. Age, gender, and mean FD were included as nuisance covariates. The Pearson correlation coefficient (*r*) and mean squared error (MSE) were calculated between actual and predicted scores for overall predictive performance. Permutation tests were carried out to assess statistical significance of correlation coefficient and MSE (randomly shuffle HDRS_%change_ scores 1,000 times). The result was reported with a cluster‐size threshold of 100 voxels. Meanwhile, rACC seed‐based FC maps were also entered into the elastic‐net prediction model to predict sham aiTBS treatment outcome.

In addition, previous evidence indicated that the initial severity of depression may affect antidepressant benefits (Kirsch et al., 2008). In order to diminish the possible influence of baseline depression severity, we further investigated whether the GBC/rACC seed‐based FC were predictive of HDRS_baseline_ scores.

## 3 RESULTS

### 3.1 Clinical data

The HDRS scores were acquired before and after of sham aiTBS treatment in 22 TRD patients (mean HDRS_baseline_ ± SD: 22.23 ± 5.99; mean HDRS_post_ ± SD: 19.32 ± 5.00; mean HDRS_%change_ ± SD: 9.31% ± 26.04%, Cohen's *d* = .358; Table 1). Paired T‐tests showed that HDRS scores prestimulation and poststimulation were statistically different (*p* = .015).

**Table 1:** Demographic information and behavioral results

	All	Males	Females	*p* value
# Subjects	22	6	16	0.033^a^
Age (years)	43 (12.24)	46.17 (13.20)	42.31 (12.14)	0.55^b^
HDRS_baseline_	22.23 (5.99)	22.50 (6.38)	22.13 (6.05)	0.90^b^
HDRS_post_	19.32 (5.00)	19.33 (6.44)	19.31 (4.60)	0.99^b^
HDRS_%change_	9.31 (26.04)	12.74 (19.19)	8.02 (28.63)	0.71^b^

Means were reported with their standard deviation in parentheses for the age and HDRS scores. *p* value: males versus females.

a Chi‐squared test.

b Independent samples *t*‐test (two‐tailed).

### 3.2 Prediction of sham‐aiTBS responses

**Figure 2:**
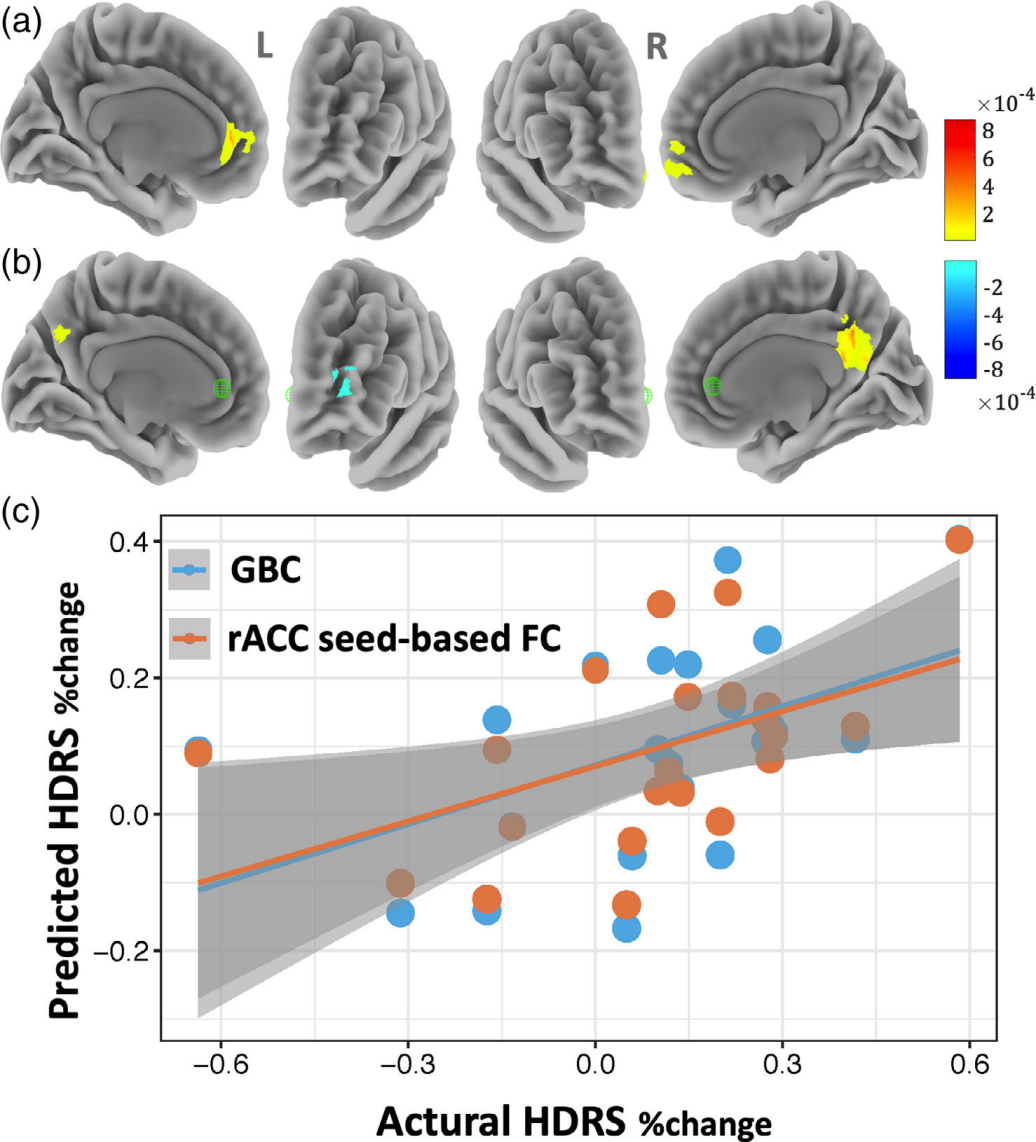
Pretreatment brain connectivity (aCompCor noise removal approach) prediction of the placebo response to sham aiTBS treatment. (a/b) Voxel weight values of multivariate elastic‐net regression, with cluster size >100 voxels; a: GBC, b: rACC seed‐based FC. (c) The predicted HDRS_%change_ scores were significantly correlated with the actual scores (GBC: *r* = .486, *p* = .023; rACC seed‐based FC: *r* = .498, *p* = .018), with age, gender, and mean FD as the nuisance covariates

We only report here the result from the aCompcor based denoising scheme for FC analysis (Figure 2), the result from the whole brain regression‐based noise correction for FC analysis are largely similar (see Figure S1). As shown in Figure 2c, the GBC predicted HDRS_%change_ scores correlated significantly with the actual scores (*r* = .486, *p* = .023; MSE = 0.050, *p* = .022). The strongest predictions of the placebo effect were located in the rostral anterior cingulate cortex/ventral medial prefrontal cortex (rACC/vmPFC; Peak MNI coordinate: [−4, 48, 0]; Figure 2a). In addition, the GBC maps were not predictive of the HDRS_baseline_ scores (*r* = .131, *p* = .562; MSE = 34.38, *p* = .562).

The rACC seed‐based FC analysis also revealed the following functional connectivity patterns contributing to the sham aiTBS response (*r* = .498, *p* = .018; MSE = 0.048, *p* = .020): a positive rsFC with precuneus/posterior cingulate (PCun/PCC) cortex and a negative rsFC with the middle frontal gyrus (Figure 2b). Analogously, the rACC seed‐based FC maps were not predictive of the HDRS_baseline_ scores (*r* = .239, *p* = .283; MSE = 32.805, *p* = .283).

## 4 DISCUSSION

In the assumption that the convergence between data‐driven and hypothesis‐dependent analysis could provide added confidence in the rACC connectivity implicated in the placebo response to sham aiTBS treatment in this clinically (treatment‐resistant) depressed sample, we used specific brain analytic methods. Traditional rsFC analysis requires a prior specification of seed ROIs, which may increase the risk of false‐positive due to multiple comparisons when multiple seeds were explored. To overcome the problem of seed specification, here we used a graph theory‐based approach (GBC) to characterize the strength of connectivity between a given voxel and the rest of the brain (Martuzzi et al., 2011). We found that the clinical placebo response to four succeeding days of sham aiTBS treatment can be predicted by the baseline GBC of the rACC/vmPFC, and by rACC seed FC with the PCun/PCC and middle frontal gyrus. These results are consistent with previous antidepressant pharmacotherapy trials showing that pretreatment rACC baseline FC predicted depression symptom improvement in placebo‐treated patients.

The predictive role of the global connectivity strength of rACC/vmPFC (DMN regions) supports the assumption that the prefrontal regions may be a critically involved in the placebo response (Benedetti, 2010; Benedetti, Carlino, & Pollo, 2011; Krummenacher, Candia, Folkers, Schedlowski, & Schönbächler, 2010). As Benedetti states, “No prefrontal control, no placebo response” (Benedetti, 2010). Especially the rACC/vmPFC regions are rich in opioid and dopamine receptors and these brain regions **—** irrespective of the form of placebo application and clinical condition — have consistently been shown to be engaged by placebo treatments (Colagiuri, Schenk, Kessler, Dorsey, & Colloca, 2015; Geuter, Koban, & Wager, 2017). For example, some recent studies examining intrinsic connectivity networks indicated that the rACC FC predicted the pharmacological‐placebo effects in depression and the placebo analgesia in chronic pain (Meyer, Yuen, Saase, & Kalisch, 2019; Sikora et al., 2016; Tétreault et al., 2016). Although further investigation is still required, our current findings extend the rACC FC as a potential biomarker of the placebo response of sham rTMS treatment. Furthermore, some recent meta‐analyses attributed a central role of the vmPFC in the placebo response (Ashar et al., 2017; Geuter et al., 2017). The vmPFC‐centric appraisal system, associated with the DMN, has been thought to be instrumental in forming social and self‐referential cognitions, conceptual expectations, cognitive beliefs, valuation, and their interactions. Based on these concepts, Ashar, Chang and Wager argued that placebos may act by engaging these brain systems to govern how a person evaluates the treatment context and ultimately influence appraisals of future well‐being (Ashar et al., 2017). Given the association with clinical improvement, we contended that a patient's functional neural correlates of appraisal processes might be important predictors of placebo responses to the sham aiTBS treatment.

We also observed that larger placebo responses for sham aiTBS were predicted by stronger baseline intra‐DMN (between the rACC and PCun/PCC) and DMN‐frontoparietal control network (FPN) rsFC (between the rACC and middle frontal gyrus). The identified rACC seed‐based FC patterns can also be linked with the hallmark symptoms of depression. Specifically, hyperconnectivity within the DMN could account for the maladaptive depressive rumination and the excessive self‐focus processing (Broyd et al., 2009; Hamilton et al., 2011; Holtzheimer & Mayberg, 2011). Hyperconnectivity between the DMN and the FPN regions may be more associated with impairments in goal‐directed behavior which is biased towards internal self‐referential thoughts (Kaiser, Andrews‐Hanna, Wager, & Pizzagalli, 2015; Whitton et al., 2018). Furthermore, Grimm et al. (2009, 2011) associated increased self‐focus with the cortical midline structures (CMS), and Northoff et al. showed an association between increased self‐focus and the CMS when clinically depressed (Northoff, 2007, 2016; Northoff, Wiebking, Feinberg, & Panksepp, 2011). Because, in our study, hyperconnectivity within the DMN and between the DMN and FPN were associated with illness severity changes (HDRS_%change_), our findings suggest that patients hyperconnectivity between these brain regions may respond stronger to sham neurostimulation treatment. See also supplemental material (Figure S2). This is consistent with the observed hyperconnectivity within the DMN as positive outcome predictor for rTMS in depression (Liston et al., 2014). Moreover, a recent emotion processing study found that DMN suppression may actually predict early unspecific treatment factors, such as placebo responses (Spies et al., 2017).

Interestingly, the rACC/vmPFC and its connectivity within the DMN have also shown promise for the prediction of the beneficial effects of active antidepressant treatment (Drysdale et al., 2017; Philip et al., 2018; Pizzagalli, 2011; Posner et al., 2013; Spies et al., 2017). A trivial explanation could be that clinical improvements resulting from accelerated stimulation protocols may partly be explained by placebo effects, which can enhance the active treatment's inherent therapeutic benefit (Baeken et al., 2013; Duprat et al., 2016; Razza et al., 2018). This corroborates with the overall effects of active pharmacotherapy and psychotherapy in the treatment of depression, which can also be decomposed into treatment‐specific effects, placebo effects, and nonspecific effects, for example, spontaneous remission (Cuijpers et al., 2012; Khan, Faucett, Lichtenberg, Kirsch, & Brown, 2012). On the other hand, although performed in healthy subjects, it has been suggested that this network‐specific increase in meso‐cortico‐limbic network connectivity may reflect a rTMS mechanism independent of subjective expectancy and placebo effects given that it was observed only shortly after active stimulation (Tik et al., 2017). However, here we found hyperconnectivity patterns within these rACC/vmPFC areas associated with placebo neurostimulation and depression severity symptom improvement, but in the depressed state.

Since we primarily focused on the application of only four succeeding days of sham stimulation an important limitation is the lack of follow‐up data on long‐term effects. Therefore, it's not yet clear whether the predictive value of baseline rsFC for prompt clinical placebo effects can be carried over for the prediction of more sustained responses over longer periods of time. Our study is also limited using a single measurement (percent reduction in HDRS scores) to provide an indication of a rather broad definition of clinical response. Future work with multiple and complementary assessments in the evaluation of the therapeutic response should address this question (Kragel et al., 2018). Of course, the interpretation of the current findings should be limited to accelerated neurostimulation protocols in depressed patients. Finally, the GBC and seed‐based FC findings in rACC must be considered as exploratory and should be verified in larger independent samples. On the other hand, we must emphasize that patients were randomly allocated to receive sham or active aiTBS and that all were naïve to the rTMS method. Therefore, this study cannot be considered just as an open pre‐post sham aiTBS study.

## 5 CONCLUSIONS

We used a novel statistical learning technique to identify the baseline rsFC biomarker for sham aiTBS treatment. Stronger intra‐DMN and DMN‐FPN rsFC predicted larger placebo responses to a therapeutic trial of sham aiTBS. These findings substantiate earlier neurobiological research, indicating that placebo responses in depression are related to (re)appraisal and changes in depressive rumination processes. Besides that sham neurostimulation may result in clinically meaningful effects, more research is needed to understand how placebo responses may influence active (accelerated) rTMS protocols.

## CONFLICT OF INTEREST

The authors declare that they have no conflict of interest.

## Supporting information


**Figure S1** Pretreatment brain connectivity (whole brain signal regression noise removal approach) prediction of the placebo response to sham aiTBS treatment. (a/b) Voxel weight values of multivariate elastic‐net regression, with cluster size >100 voxels; a: GBC, b: rACC seed‐based FC. (c) The predicted HDRS_%change_ scores were significantly correlated with the actual scores (GBC: *r* = .522, *p* = .013; rACC seed‐based FC: *r* = .498, *p* = .018), with age, gender, and mean FD as the nuisance covariates.
**Figure S2.** One‐sample t‐tests results of the rACC seed‐based FC under pre and post sham aiTBS (*p* < .05, FWE correction at the cluster level with a voxel‐level threshold of *p* < .001 uncorrected). Two confound regression strategies were used: aCompcor, and global signal regression (GSR). We arbitrary have split the change HDRS score into two groups yielding a group with low mean split response (*n* = 8) and a group with high mean split response (*n* = 14). Mean HDRS_%change_ = 9.31%.

## Data Availability

The data used in this study were collected in the University Hospital, Ghent (Ghent University). When they were collected, no explicit agreement to sharing was requested to the subjects, so it's unfortunately impossible to publicly share them on a third‐party repository. The code used in this study is available on NITRC (CONN, version 18.b, http://www.nitrc.org/projects/conn), and on scikit‐learn (version 0.19.0, https://scikit-learn.org/stable/index.html).
