# Supplementary material for: Baseline functional connectivity may predict placebo responses to accelerated rTMS treatment in major depression
Source: Hum Brain Mapp. 2019 Oct 21;41(3):632–9. doi: 10.1002/hbm.24828 (PMC7267925; doi:10.1002/hbm.24828)
Supplement: Supplementary file 1 — Figure S1 Pretreatment brain connectivity (whole brain signal regression noise removal approach) prediction of the placebo response to sham aiTBS treatment. (a/b) Voxel weight values of multivariate elastic‐net regression, with cluster size >100 voxels; a: GBC, b: rACC seed‐based FC. (c) The predicted HDRS%change scores were significantly correlated with the actual scores (GBC: r = .522, p = .013; rACC seed‐based FC: r = .498, p = .018), with age, gender, and mean FD as the nuisance covariates. Figure S2. One‐sample t‐tests results of the rACC seed‐based FC under pre and post sham aiTBS (p < .05, FWE correction at the cluster level with a voxel‐level threshold of p < .001 uncorrected). Two confound regression strategies were used: aCompcor, and global signal regression (GSR). We arbitrary have split the change HDRS score into two groups yielding a group with low mean split response (n = 8) and a group with high mean split response (n = 14). Mean HDRS%change = 9.31%. [file HBM-41-632-s001.docx]

**
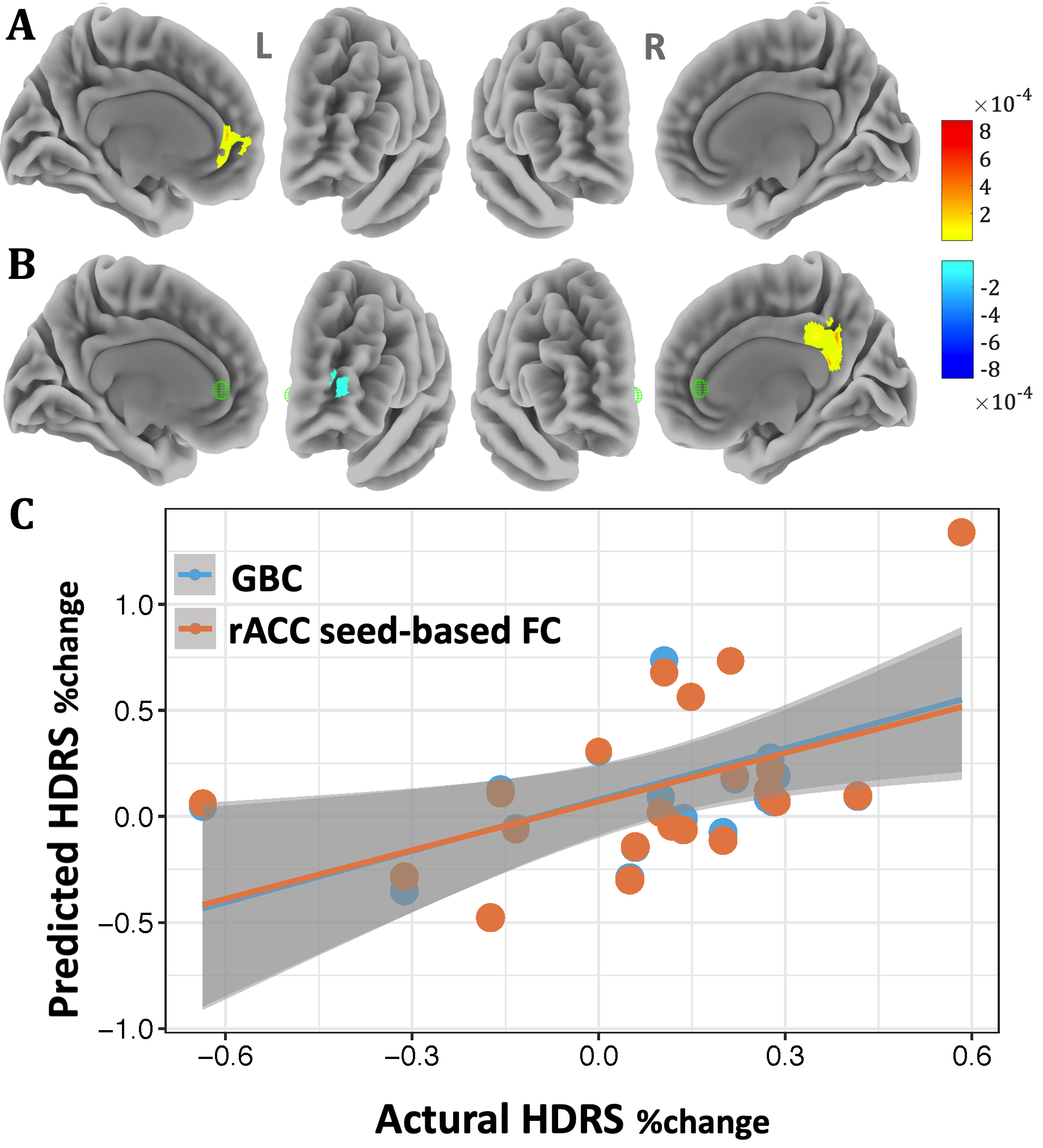
**

**Figure S1.** Pretreatment brain connectivity (whole brain signal regression noise removal approach) prediction of the placebo response to sham aiTBS treatment. (A/B) Voxel weight values of multivariate elastic-net regression, with cluster size > 100 voxels; A: GBC, B: rACC seed-based FC. (C) The predicted HDRS_%change_ scores were significantly correlated with the actual scores (GBC: $r$= 0.522, $p$= 0.013; rACC seed-based FC: $r$= 0.498, $p$= 0.018), with age, gender, and mean FD as the nuisance covariates.


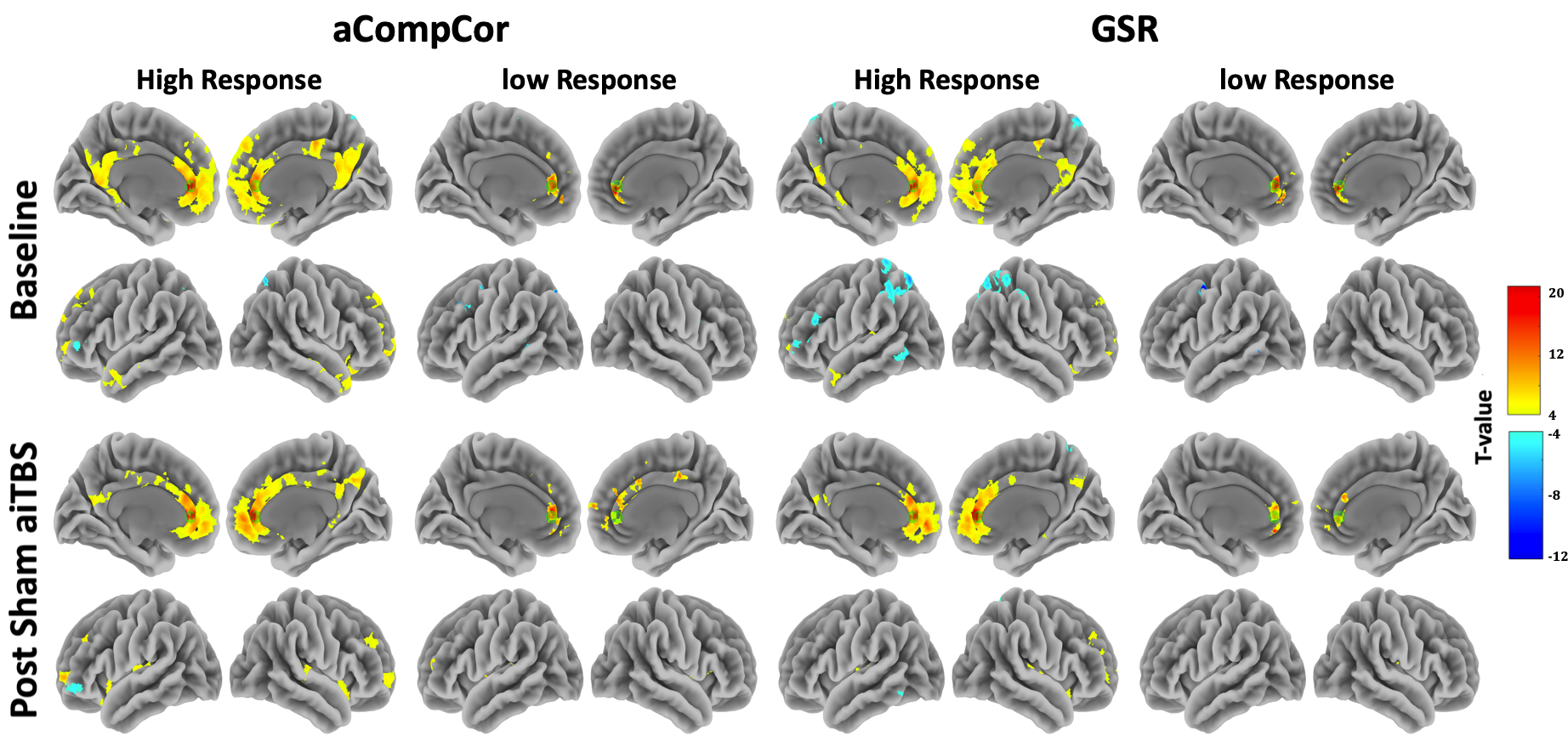


**Figure S2.** One-sample t-tests results of the rACC seed-based FC under pre and post sham aiTBS (*p* < 0.05, FWE correction at the cluster level with a voxel-level threshold of *p* < 0.001 uncorrected). Two confound regression strategies were used: aCompcor, and global signal regression (GSR). We arbitrary have split the change HDRS score in two groups yielding a group with low mean split response (*n*=8) and a group with high mean split response (*n*=14). Mean HDRS_%change_ = 9.31%.
